# A Case of Cementless Impaction Bone Graft in a Revision Total Hip Arthroplasty Requiring Calcar Reconstruction

**DOI:** 10.1155/2021/8811593

**Published:** 2021-02-27

**Authors:** Shigeo Ishiguro, Kunihiro Asanuma, Tatsuya Tamaki, Kazuhiro Oinuma, Akihiro Sudo

**Affiliations:** ^1^Orthopaedic Surgery, Kameyama Medical Center, Mie, Japan; ^2^Department of Orthopaedic Surgery, Mie University Graduate School of Medicine, Mie, Japan; ^3^Funabashi Orthopaedic Hospital, Chiba, Japan

## Abstract

**Introduction:**

In cases of bone deficiency or osteoporosis, and especially in revision cases, there were only two options for treatment until the impaction bone graft procedure was proposed. These were cemented or cementless femoral prosthesis. In the early 1990s, the use of impaction bone graft with a cemented mantle had gained popularity and had proven to be clinically effective. In Germany, a cementless impaction bone graft procedure using Corail® (DePuy Synthes) stems was devised, and functional scores were similar to conventional cemented Impaction bone grafts. *Case presentation*. A 48-year-old man presented with femur loosening of a reamed bipolar arthroplasty performed in 1990. The patient was treated with a cementless impaction bone graft using a Corail® (DePuy Synthes) stem in the femur in revision THA surgery, and the calcar was reconstructed by allograft.

**Results:**

At five years, the calcar allograft united with the host bone, and the femoral component showed no subsidence.

**Conclusion:**

Calcar reconstruction with a strut allograft, aimed at preventing sinking of the stem was key in this operation. Surgical indication for femoral cementless impaction bone graft should be for loosened femoral prosthesis in a type II Paprosky classification, where only the cortical bone of the isthmus is partially affected, cortical thinning does not exist, and it is mechanically strong enough for the allograft tip impaction. The procedure was safely feasible through the direct anterior approach.

## 1. Introduction

In cases of bone deficiency or osteoporosis, and especially in revision cases, there were previously only limited options for treatment until the impaction bone graft procedure was proposed. Among the limited options, the two major options were whether the femoral prosthesis should have cemented or cementless proximal femoral fixation. In the early 1990s, the use of impaction bone graft with a cemented mantle had gained popularity and had proven to be clinically effective and histologically valid [1]. Some clinicians hold the view that conventional cemented impaction bone graft is not ideal for the preservation of bone stock [2], and after 2000, some authors began to report the use of cementless impaction bone grafting using a long stem, with the aim of preserving bone stock [3]. Several authors compared conventional cemented impaction bone grafting using polished Landos Fjord-CrCo stems, and cementless impaction bone grafting using Corail® (DePuy Synthes) stems, and reported that the functional scores were similar [4].

## 2. Case History

A 48-year-old male had been the recipient of a reamed bipolar hemiarthroplasty using the conventional posterior approach 25 years prior, following secondary osteoarthritis due to synovectomy for pigmented villonodular synovitis. For the preceding 15 years, he had encountered thigh pain, leg length discrepancy, and groin pain when he took his first few steps upon standing.

**Figure 1:**
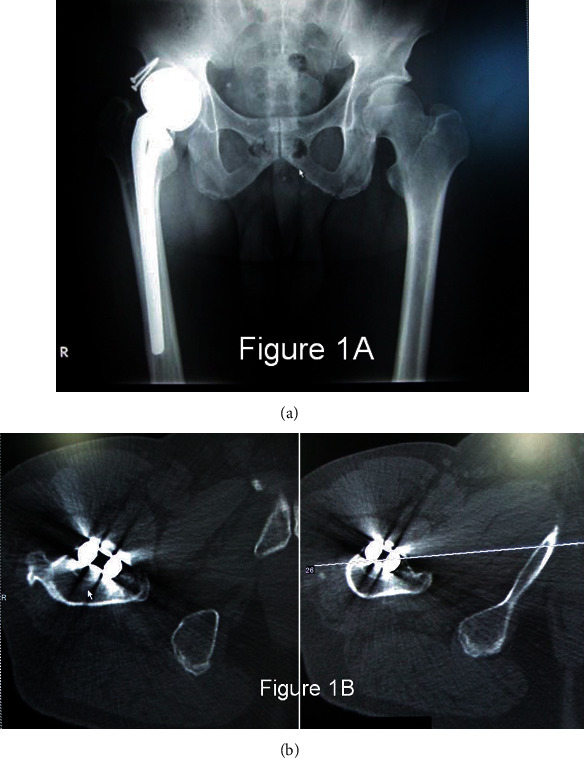
(a) Plain X-P shows the femoral prosthesis subsidence and slight internal migration of bipolar head. (b) Preoperative CT shows the retroverted femoral prosthesis and calcar bone absorption.

In 2015, he visited our institution for surgical intervention. His Harris hip score was 42.8 points. Plain X-rays showed severe subsidence of the right femur, resulting in a 2 cm leg discrepancy (Figure 1), and this case was classified type II according to the Paprosky classification (Table 1). A CT scan showed a 30-degree retroverted femoral prosthesis, seemingly caused by the subsidence [5] of the prosthesis (Figure 1). His preoperative CRP value was negative, and his clinical diagnosis was aseptic loosening.

On day 2 in the hospital, the revision surgery was performed using the direct anterior approach without the use of a traction table. The loosened femoral prosthesis with bipolar head was easily removed. After AO screw removal, the acetabular component was fixed. The surrounding tissue in the femoral canal was then curetted. The removed femoral prosthesis was an Osteonics Moor-type femoral stem collared prosthesis. The cementless impaction bone graft procedure began. Unlike an ordinary cemented Impaction bone graft, distal occlusion of the femur was not performed as the planned implant was shorter than the one which was removed in length, and the isthmus level showed sclerosis in preoperative X-rays. A guide wire was not used, and the impaction bone graft procedure was done manually by ordinary Corail broach. After the impaction bone graft, the size of the femoral prosthesis was 3 sizes smaller than what was appropriate to fill or occupy the femoral cavity using a trial prosthesis without a bone graft. The final femoral broaching showed no movement when it was hammered distal-ward and this was confirmed by intraoperative X-ray (image not shown). Though the Corail prosthesis was mechanically well-fixed intraoperatively after the impaction graft of morselized cancellous bone derived from one femoral head allograft, a calcar deficient area 10 mm in height was still present. Hence, before the final hammering of the implant prosthesis, a strut allograft was placed on the lesser trochanter with ultrahigh molecular weight polyethylene (UHMWPE) tape (NESPLON Cable System, Alfresa Pharma Corporation, Osaka, Japan). The tape was passed through the equipped passer, around the femur, above the lesser trochanter level. In cases requiring bone graft to the calcar area, calcar allograft is meticulously contoured by Rewell, and a shallow gutter is made by bone-saw in order to prevent deviation of the tape. In this case, however, it was not necessary, as the allograft was well stacked in the desired area below the collar of the Corail stem. After final implantation of the femoral prosthesis, the NESPLON Cable System was tightened and settings finalized. The operation time was 2 hours and 48 minutes. Intraoperative bleeding was 910 ml, and transfusion was not necessary due to the intraoperative blood salvage procedure.

On postoperative day one, the patient was allowed to bear weight as tolerated with an assistive cane. On day four, the patient was discharged from hospital, and the next day returned to sedentary work.

**Figure 2:**
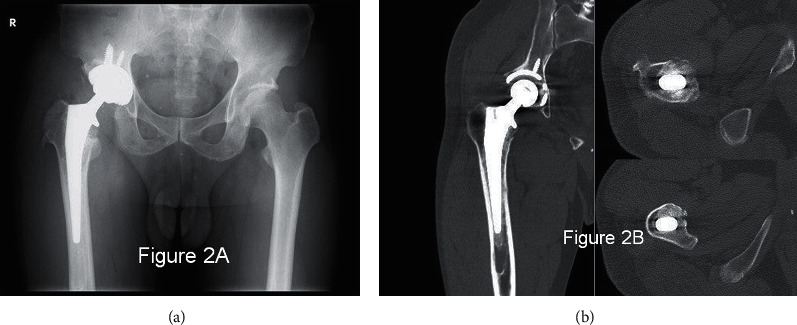
(a) Postoperative X-ray shows the strut allograft above the lesser trochanter and densely impacted bone graft in the thigh. (b) CT at 10 weeks shows regional welding between femoral prosthesis and femoral cortex especially on the lesser trochanter level. Beneath the lesser trochanter, a 4.5 cm long longitudinal linear fracture line, which had already been in the process of healing was observed (not all images shown).

Postoperative X-rays show densely impacted cancellous bone inside the femur, the prosthesis appeared stable with no subsidence, and CT scans at 10 weeks depicted a biomechanically well-fixed femoral prosthesis (Figure 2). The patient was allowed to bear weight as tolerated from this point.

**Figure 3:**
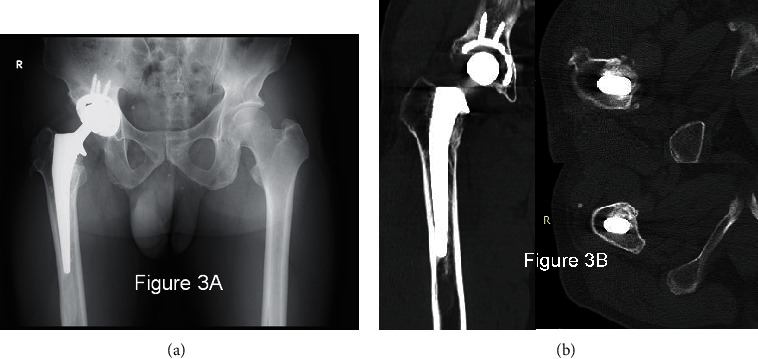
(a) 2-year postoperative X-ray shows minute subsidence and homogenized allograft on the calcar. (b) CT at 2 years proves rigid fixation in Gruen zones 1, 2, and ongoing osteogeneration in other zones.

**Figure 4:**
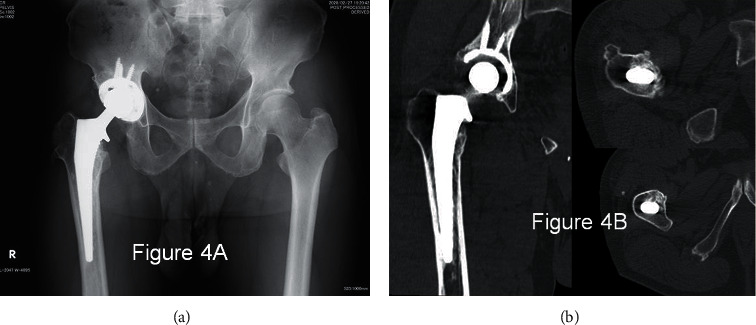
(a) 5 years and 3 months have passed and radiological images (Figure 4) showed that the calcar allograft united to the host bone with partial absorption and it showed no further subsidence compared to 1-year X-ray. (b) CT at 5 years and 6 months proves rigid fixation in all Gruen zones except in zone 1.

Radiological images (Figure 3) at 1 year indicated more rigid fixation in Gruen zones 1 and 2 than was indicated at 10 weeks, and it appeared that bone conduction had occurred in the calcar reconstructed by the allograft. At 5 years, radiological images (Figure 4) showed that the calcar allograft had united to the host bone accompanying partial absorption, and it showed no further subsidence compared to the 1-year X-ray.

## 3. Discussion

To allow rapid recovery, rigid fixation was a prerequisite, and bone stock preservation was also desirable. To meet the preference of avoiding cement usage, this procedure was a reasonable option.

In choosing cementless stem revision, there may have been discussion that the situation could have been better handled with a distally fixed stem such as a cylindrical fullcoat stem, or cone/conical stem construct, without the need for bone grafting. With the aim of bone stock preservation, our choice was justified because stress shielding expected on the proximal femur can be theoretically minimized. Weeden and Paprosky found that patients with type II or IIIa defects had a 5% failure rate in cylindrical, extensively porous coated stems at 14 years [6]. This rate does not seem acceptable in young patients who are expected to live long enough to have concerns about revision surgeries.

This case had several other treatment options including a proximal femoral strut allograft, cementless fixation with a modular tapered flute stem [7], and cementless impaction grafting using a modular long stem, as is the recent trend [8]. The obvious advantages to proximal femoral allograft are the ability to restore bone stock, particularly in younger patients, and the provision of a biologic anchor for the adductor complex [8].

To allow rigid cementless fixation in cases of femoral defects (type I-III as described by Paprosky) [7], Wimmer et al. conducted a retrospective survey of procedures done both with and without cementless impaction bone graft in proximal femur reconstruction combined with longer stem usage to obtain distal diaphyseal stem fixation. They concluded that cases with cementless impaction bone graft showed better radiological and clinical results. Their method, however, can neither preserve distal bone stock nor hold an allograft tip on the medial aspect of the calcar, which is important for the precise adjustment of limb length [9].

In terms of biomechanical properties, densely packed particulate grafts also have been used for the structural support of implants [10]. We clinically applied this concept for the diapyseal fixation of the Corail stem. As this case was classified type II according to Paprosky, only the cortical bone of the isthmus was partially affected by the tip of the loose stem in the varus position, and cortical thinning did not exist in the diaphysis. We had assumed that sufficient allograft chip would be possible to be densely packed into the femoral canal to secure rigid fixation [10]. With conventional cementless revision arthroplasty, it would have been necessary to choose a larger diameter stem, which may cause residual thigh pain [11].

Several authors reported that intraoperative fracture occurred in 2 to 24% of revision THAs with tapered fluted stems [12–14], the wide range depending upon the report cited, and likely depending upon the diagnostic method used. This case was also complicated by intraoperatively unrecognized fissure fracture, which must have been caused by the bone packing procedure. However, both the strut allograft on the calcar and the femur were tightly cerclaged with Nesplon tape, and therefore, this caused no clinical symptoms. In comparing cementless impaction bone graft using a distally fixed stem in revision THA, Wimmer et al. described that intraoperative shaft fissure occurred in 5% of procedures, and postoperative subsidence occurred in 8%. Subsidence of 2 mm or more occurred in 4% at 4.4 ± 1.8 years follow-up [3]. If recognized, fissure of the shaft is conservatively treatable, to allow early ambulation with or without the necessity of the calcar allograft. The area above the lesser trochanter level should be prophylactically cerclaged for safety as vigorous packing of the canal can produce a fissure or split.

Paprosky et al. indicated that for revision THA cases with altered lesser trochanter anatomy, the use of a modular tapered stem is advised as it offers the advantages of accurately controlling femoral version and length [8]. The downside of this, however, includes implant fracture and corrosion that causes the surgeon to revise the components [7, 15]. Hence, we judged that a modular tapered stem was not suitable for this case, as the patient is young and active.

In a search of English literature, cementless impaction bone graft in revision THA is scarce [4, 12], and there is only one English abstract described by German authors, randomizing Paprosky grade II patients into two groups intraoperatively, and comparing cementless impaction grafting using Corail stems with cemented impaction bone grafting using polished femoral stems. [4]. They concluded that functional scores and bone density around the femoral stem at up to five years were similar. Their data did not provide other radiological findings such as stem sinking, interface change between the implant and the surrounding femur, or the fate of the allograft tip. Furthermore, no patients in their two series received calcar reconstruction with a strut allograft, which is supposed to prevent sinking of the stem.

Osteointegration toward the hydroxyapatite coating on the bottom of the collar of the Corail prosthesis has actually occurred as we expected [16]. Further clinical and radiological observation, however, will be necessary.

We conclude that our method of cementless impaction bone grafting using a Corail stem may be an option in the next generation of procedures in cases concerning relatively young patients whose bone quality is not poor, and whose life expectancy is long enough to expect possible revision.

Consent for publication has been obtained from the patient.

This research received no specific grant from any funding agency in the public, commercial, or not-for-profit sectors. The authors declare that there is no conflict of interest.

## 4. Summary

The present case report suggests that cementless impaction bone grafting and strut calcar allograft using a Corail stem can be indicated for Paprosky stage II femoral deficiency with calcar deficiency in a young active patient whose femoral cortex is strong enough to tolerate a cementless impaction bone grafting procedure.

## Figures and Tables

**Table 1:** Paprosky classification of femoral deficiency in rev1s1 on total hip replacement.

Type	Description
I	Minimal metaphyseal bone loss, intact diaphysis
II	Extensive metaphyseal bone loss, intact diaphysis
IIIa	Extensive metaphyseal bone loss, diaphyseal bone loss with >4 cm of scratch fit at the isthmus
IIIb	Extensive metaphyseal bone loss, diaphyseal bone loss with <4 cm of scratch fit at the isthmus
IV	Extensive metaphyseal bone loss and nonsupportive diaphysis

## Data Availability

The [DATA TYPE] data used to support the findings of this study are included within the article.
